# One-year results of a novel self-adhesive bulk-fill restorative and a conventional bulk-fill composite in class II cavities—a randomized clinical split-mouth study

**DOI:** 10.1007/s00784-021-04019-y

**Published:** 2021-06-15

**Authors:** Fabian Cieplik, Konstantin J. Scholz, Julian C. Anthony, Isabelle Tabenski, Sarah Ettenberger, Karl-Anton Hiller, Wolfgang Buchalla, Marianne Federlin

**Affiliations:** Department of Conservative Dentistry and Periodontology, University Hospital Regensburg, Franz-Josef-Strauß-Allee 11, 93053 Regensburg, Germany

**Keywords:** Class II, Filtek one, Self-adhesive, RBC, Bulk-fill

## Abstract

**Objectives:**

In the context of the phase-down of amalgam, development of easily applicable, permanent restorative materials is of high clinical interest. Aim of this study was to evaluate the clinical performance of a novel, tooth-colored, self-adhesive bulk-fill restorative (SABF, 3M Oral Care) and a conventional bulk-fill composite (Filtek One, 3M Oral Care; FOBF) for restoring class II cavities. The null-hypothesis tested was that both materials perform similar regarding clinical performance.

**Materials and methods:**

In this randomized split-mouth study, 30 patients received one SABF and one FOBF restoration each. Scotchbond Universal (3M Oral Care) was used as adhesive for FOBF (self-etch mode), while SABF was applied directly without adhesive. Restorations were evaluated by two blinded examiners at baseline, 6 months and 12 months employing FDI criteria. Non-parametric statistical analyses and *χ*^2^-tests (*α* = 0.05) were applied.

**Results:**

Thirty patients (60 restorations) were available for the 6- and 12-month recalls exhibiting 100% restoration survival. All restorations revealed clinically acceptable FDI scores at all time points and for all criteria. Only regarding esthetic properties, FOBF performed significantly better than SABF regarding *surface lustre* (A1) and *color match and translucency* (A3) at all time points and *marginal staining* (A2b) at 12 months.

**Conclusions:**

The null-hypothesis could not be rejected. Both materials performed similarly regarding clinical performance within the first year of clinical service. SABF exhibited slightly inferior, but clinically fully acceptable esthetic properties as compared to FOBF.

**Clinical relevance:**

Within the limitations of this study, the self-adhesive bulk-fill restorative showed promising results and may be recommended for clinical use.

## Introduction

Tooth-colored resin-based composite (RBC) materials have been extensively employed for the restoration of posterior teeth for more than three decades and can be regarded as first-choice direct restoratives in contemporary dentistry [1–4]. RBCs can achieve high clinical longevity for posterior restorations with annual failure rates of about 1.7% over 12 years [5], or even 1.1% to 1.55% over 30 years [6], similar to amalgam restorations [5]. Following the Minamata Convention on Mercury and the global amalgam “phase-down” program, there is increasing demand for restorative materials with high clinical longevity that are cost-effective, easy-to-use with low technique sensitivity, and can serve as true alternatives to amalgam [7–9].

In this context, bulk-fill RBCs, which can be placed in increments of up to 5 mm, have been introduced to the market as an approach to facilitate clinical handling by skipping the time-consuming incremental application technique and reducing technique sensitivity [9, 10]. Bulk-fill RBCs are available as flowable bulk-fill restoratives and as high-viscosity, sculptable materials, which are both characterized by an increased depth of cure and reduced polymerization shrinkage as compared to conventional RBCs [11]. Flowable bulk-fill RBCs usually need to be covered by a final composite “capping” layer due to their reduced wear resistance, whereas this additional “capping” step is not necessary for high-viscosity bulk-fill RBCs [12]. High-viscosity bulk-fill RBCs further present higher depth of cure and similar or lower volumetric shrinkage as compared to conventional RBCs in vitro [13–15]. Although high-viscosity bulk-fill RBCs offer considerable ease in handling as compared to conventional RBCs, they still require preceding application of a separate adhesive system [10].

In this context, further reduction of treatment steps in terms of self-adhesion is an interesting aspect. Self-adhesive resin cements have been widely investigated in vitro [16, 17] as well as in vivo [18, 19], and thus can be considered clinically established materials for luting of indirect ceramic restorations [20] as well as fiber glass posts [21]. On this basis, flowable self-adhesive RBCs have been developed a few years ago and have been investigated in several in vitro studies [22–25]. They showed inferior shear bond strength [24, 25], more interfacial defects in dentin [22], and less micromorphological interactions with smear-covered tooth substances [23] as compared to conventional flowable RBCs used with a separately applied adhesive system. Accordingly, a clinical trial showed unacceptable performance of a flowable self-adhesive RBC for restoration of non-carious cervical lesions after clinical service of 6 months, mostly due to loss of retention [26]. However, novel self-adhesive RBCs are currently being developed and marketed by different companies, which may have better mechanical properties and thus may fulfill higher clinical restorative demands, also for stress-bearing areas such as in class II restorations [9, 27].

For serving as a true alternative to amalgam, a restorative material should ideally combine bulk-fill and self-adhesive properties to avoid the additional use of an adhesive system or the necessity of a retentive and thus invasive cavity preparation [27, 28]. The novel self-adhesive bulk-fill restorative (SABF; 3M Oral Care, St. Paul, MN, USA) is a tooth-colored, dual-curing, self-adhesive, resin-based bulk-fill restorative material that does not require retentive cavity preparations, conditioning of dental hard tissues or separate application of an adhesive, and can be placed in one bulk with unlimited depth of cure, as specified by the manufacturer.

The aim of this randomized controlled clinical split-mouth study was to evaluate the clinical performance of class II restorations placed with SABF or a conventional bulk-fill composite (Filtek™ One Bulk Fill, FOBF; 3M Oral Care), whereby the latter was used in combination with a universal adhesive (Scotchbond™ Universal, SBU; 3M Oral Care). The null-hypothesis tested was that both materials perform equally regarding clinical performance as evaluated by the FDI clinical criteria and scoring system [29, 30].

## Material and methods

### Test materials

A novel self-adhesive dual-curing bulk-fill material (SABF) and a conventional light-curing bulk-fill RBC (Filtek™ One Bulk Fill, FOBF) were used in this study, whereby the latter was applied in combination with a universal adhesive (Scotchbond™ Universal, SBU; all: 3M Oral Care, St. Paul, MN, USA) in self-etch mode.

SABF is a tooth-colored, dual-curing, self-adhesive, resin-based bulk-fill restorative material, consisting of a powder and a liquid part in a capsule. The powder part contains acid-reactive glass fillers; the liquid part consists of polymerizable components with an acidic moiety to promote self-adhesion. The composition further comprises a dual-cure initiator system which is distributed between the powder part and the liquid part and comprises camphorquinone as well as oxidizing and reducing agents. SABF bears the CE mark and is in compliance with all requirements outlined in ISO 4049 (Dentistry—polymer-based restorative materials) for a type 1 (polymer-based restorative materials claimed by the manufacturer as suitable for restorations involving occlusal surfaces), class 3 material (materials that are cured by the application of external energy and also have a self-curing mechanism present (“dual cure” materials)). It meets the demands for working and setting time, water sorption, solubility, shade, color stability, radiopacity, and flexural strength (> 80 MPa).

The material composition of FOBF and SABF is shown in Table 1, as specified by the manufacturer.

**Table 1:** Test materials

Component	FOBF	SABF
Neutral methacrylate monomers for network formation	Aromatic urethane dimethacrylate (AUDMA), addition-fragmentation monomer (AFM), diurethane dimethacrylate, 1,12-dodecane dimethacrylate	Crosslinking dimethacrylate, triethylene glycol dimethacrylate (TEGDMA)
Acidic methacrylate monomer for support of adhesive properties	none	Phosphoric acid functionalized methacrylate
Initiator system	Camphorquinone-based	Camphorquinone, oxidizing and reducing agents
Filler system	76% (w/w) nano-silica/zirconia filler, ytterbium trifluoride filler	74% (w/w) strontium-fluoro-alumino-silicate filler, zirconia-silica filler

Material composition of FOBF and SABF, as specified by the manufacturer

### Study design

The present study is a 1-year follow-up examination of a prospective controlled randomized clinical split-mouth study investigating the clinical performance of two restorative materials for restoration of class II cavities in premolars and molars, one being a novel self-adhesive dual-curing bulk-fill material (SABF), one a conventional light-curing bulk-fill RBC (FOBF) applied in combination with a universal adhesive (SBU) in self-etch mode. The sample size calculation was based on a previous study on the clinical success rate of flowable composites for restoration of non-carious cervical lesions (NCCLs) [31]. Assuming a type I error of 0.05, a power of 80% and a relative hazard of 0.33544, the minimum sample size was calculated to be 26 patients with two restorations each for this split-mouth study. To compensate for possible dropouts in the future, it was decided to recruit 30 patients.

The study design followed the requirements outlined in the CONSORT 2010 statement [32] and was approved by the ethics committee of the University of Regensburg (reference: 17–698-101) in accordance with the 1964 Helsinki declaration and its later amendments or comparable ethical standards. Written informed consent was obtained from all individual participants included in the study after receiving a detailed description of the proposed treatments and agreeing to participate in a strict recall program for at least 3 years with recall appointments after 6-mo, 12-mo, 24-mo, and 36-mo. The study has been registered at the German Clinical Trials Register (ref. DRKS00013564; Universal Trial Number (UTN): U1111-1206–2853).

### Patient selection

Thirty patients were recruited from the patient pool of the Department of Conservative Dentistry and Periodontology of the University Hospital Regensburg (Germany). For inclusion, patients had to be between 18 and 75 years old and in need of restorative treatment on at least two class II cavities in premolars or molars because of primary caries, secondary caries or otherwisely failed restorations. Patients were excluded if they were suffering from serious medical disorders (ASA score > 2), if they showed clinical signs of bruxism, traumatic malocclusion, if they were pregnant or breast feeding at the time of restoration placement, or if they exhibited any intolerance or allergy toward the applied restorative materials.

The teeth to be restored had to show no signs of pulpitis but positive sensitivity testing using the ice-spray test (Endo-Frost, Roeko/Coltene/Whaledent, Langenau, Germany), periodontal probing depth ≤ 5.5 mm and tooth mobility ≤ grade I (i.e., mobility of the tooth noticeable, but not visible). Only posterior teeth with class II cavities that had antagonistic contact and at least one proximal contact were included in this study. No further inclusion or exclusion criteria in terms of extension of the cavities were applied. Teeth to be restored did not have to be contralateral, and could comprise both tooth types. In case study restorations were in antagonistic contact with each other, each study restoration had to have at least one antagonistic occlusal contact to natural tooth substances.

The full-mouth papillary bleeding index (PBI) as described by Saxer and Mühlemann [33] was employed as a measure of gingival inflammation and the overall oral hygiene level and had to be 30% or less.

### Clinical restorative procedures

The restorative procedures were standardized and performed by three specially instructed and experienced general dentists (MF, JCA, SE) in the Department of Conservative Dentistry and Periodontology. Randomization of the restorations to either the control group (FOBF) or the test group (SABF) was performed by drawing a lot from an envelope assigning a material (FOBF or SABF) to the tooth with the lower FDI number and then to the tooth with the higher FDI number. Whenever possible, restorations were placed with rubber dam isolation; if no placement of rubber dam was possible, moisture control and a dry operative field were accomplished using cotton rolls, parotis pads, and a saliva ejector. Prior to isolation of teeth, the appropriate shades of FOBF or SABF were selected using a VITAPAN^®^ classical shade guide (VITA Zahnfabrik, Bad Säckingen, Germany).

The tooth surface was cleaned with a slurry of pumice and rinsed with water spray in order to remove any remaining biofilm. Then, the defective restoration or the carious lesion was removed and a class II cavity was prepared according to the extension of the original defect and without any focus on mechanical retention by using a high-speed handpiece and diamond burs under sufficient water cooling. Soft carious dentin, as detected using a dental explorer (EXS96, Hu-Friedy, Chicago, IL, USA), was removed with round carbide burs at low speed until firm dentin without any signs of bacterial infection was reached (SiroInspect^®^, Dentsply Sirona, Bensheim, Germany). If indirect pulp capping was indicated, Kerr Life™ (KaVo Kerr, Brea, CA, USA) was used. Restoration placement was performed in combination with the Hawe Blue Adapt™ sectional matrix 2752 system and Hawe Sycamore™ interdental wedges (both: KaVo Kerr). For FOBF restorations, SBU was used as an adhesive system in self-etch mode. SBU was actively applied with a disposable brush tip for 20 s, air-dried gently for 5 s creating a uniform bond thickness, and light-cured for 10 s (Satelec^®^ mini LED, Acteon, Mérignac, France; 1250 mW/cm^2^). Then, FOBF was placed in bulk of up to 4 mm. In case 4 mm was not enough to fully restore the tooth, a second layer FOBF was placed on top of the first 4-mm layer. Light-curing was performed for 20 s per bulk. For SABF restorations, SABF was mixed in a capsule-mixing device (CapMix™, 3M Oral Care) for 15 s, placed in one bulk in the unconditioned cavities, and light-cured for 20 s. The placement procedure for SABF was similar to that of known glass ionomer cements. The capsule tip was placed in the proximal box and while gradually moving the tip in a coronal direction the material was extruded, ensuring that the material adapted itself to the cavity bottom and the cavity walls. The solely light-curing FOBF allowed ample time to sculpt the material before light polymerization. Therefore, morphology of FOBF restoration was achieved by sculpting of the material in the unpolymerized condition. On the other hand, the dual-curing SABF allowed only little time for sculpting before auto-polymerization started, and thus needed to be overfilled to a certain extent and adapted to the cavity walls in an outward direction before the final restoration morphology could be achieved by subtractive measures. Finishing and polishing of the restorations from both materials was performed using fine (46 μm) and ultra-fine (25 μm) diamond burs (Hager & Meisinger, Neuss, Germany), Arkansas stones (Acurata, Thurmansbang, Germany), and the Sof-Lex™ system (Sof-Lex™ Contouring and Polishing Discs, Sof-Lex™ Pre-Polishing Spirals, Sof-Lex™ Diamond Polishing Spirals; 3M Oral Care).

### Clinical examination

Clinical examinations were performed by two blinded examiners each from a pool of examiners (FC, KJS, JCA, IT, WB, MF), who had all been calibrated in advance and who were not involved in the treatments and neither aware of the restorative material used in the individual teeth nor of earlier examination scores. The restorations were evaluated at baseline (BL; 1–2 weeks after restorative procedures) as well as after 6 months (6-mo) and 12 months (12-mo). The FDI clinical criteria and scoring system was employed for evaluation of the restorations [29, 30]. The following were selected here for evaluating the clinical performance of the restorations after up to 12 months:

- Esthetic properties
  - Surface lustre (A1)
  - Surface staining (A2a)
  - Marginal staining (A2b)
  - Color match and translucency (A3)
  - Esthetic anatomical form (A4)
- Functional properties
  - Fracture of material and retention (B5)
  - Marginal adaptation (B6)
  - Occlusal contour and wear (B7)
- Biological properties
  - Postoperative (hyper-)sensitivity and tooth vitality (C11)
  - Tooth integrity (enamel cracks, tooth fractures) (C13)
  - Periodontal response (C14)

The clinical assessment of the investigation criteria was done by means of a five-score scale [29, 30]. Tooth vitality was investigated using the ice-spray test and postoperative hypersensitivities were determined by interview of the patients. Each restoration was examined independently by both examiners. In case of any disagreement between the examiners, consensus between both examiners was reached by immediate joint reexamination and discussion with the patient still being present.

### Data analysis

For the evaluation of clinical performance over time and comparison of both materials as documented by the FDI criteria, 30 patients with both restorations under risk were available for the BL evaluation as well as for 6-mo and 12-mo recall appointments. For evaluating significant differences *between* both materials at an examination time point, or *within* a given material over time, pairwise *χ*^2^ tests were applied for each single FDI criterion on a significance level of *α* = 0.05. All statistical analyses were performed using SPSS for Windows, version 25 (SPSS Inc., Chicago, IL, USA).

## Results

### Patient characteristics

**Fig. 1:**
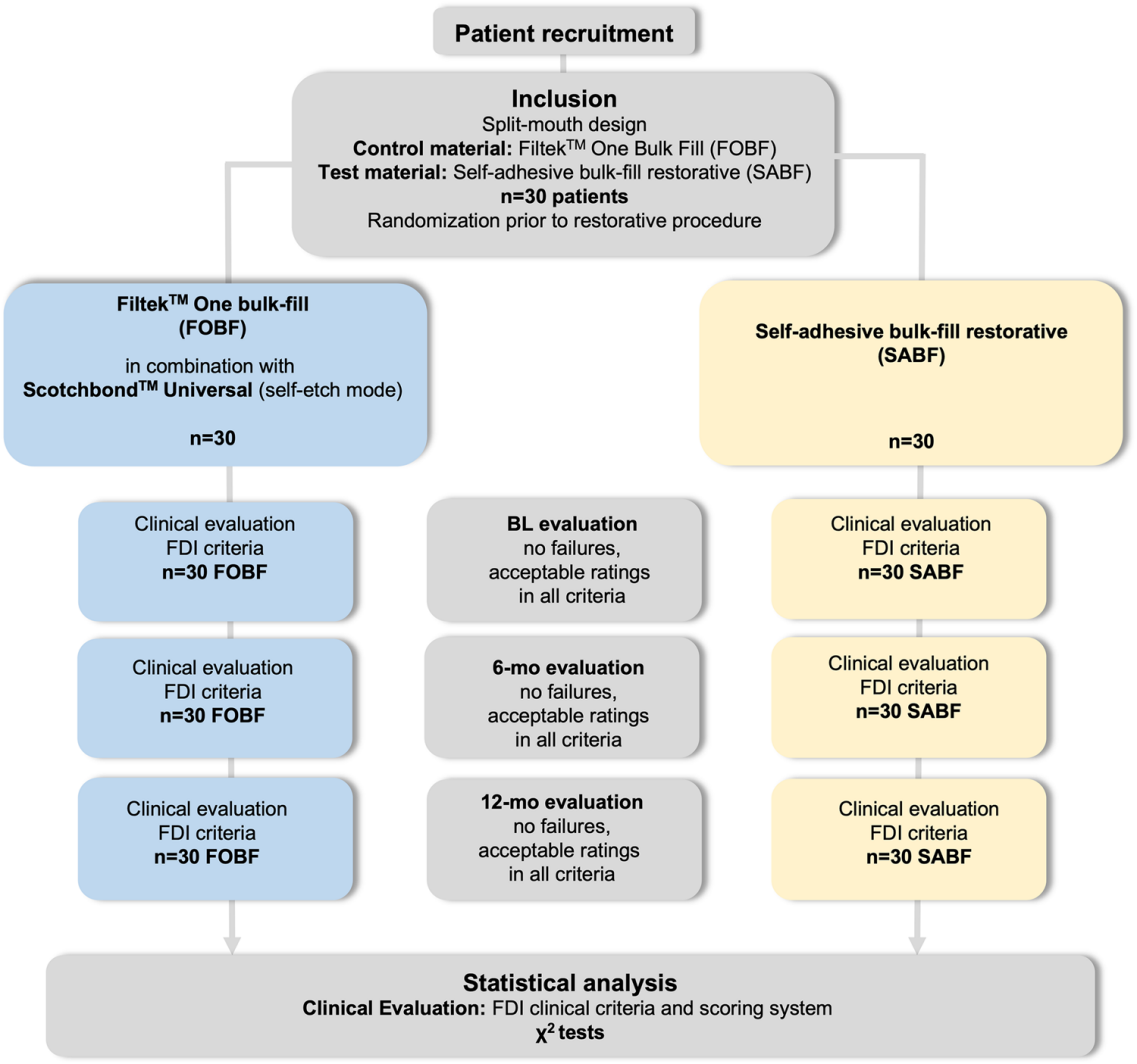
Flow of participants through the stages of this study

Thirty patients (21 females, 9 males) were included in the study. Twenty-five patients were non-smokers, 5 patients were smokers. Patient age at time of inclusion ranged from 21 to 58 years (median: 40 years). The median (1^st^; 3^rd^ quartile) full-mouth PBI was found to be 8.5% (5%; 11.5%) at BL, 8.5% (5%; 15%) at 6-mo, and 7.5% (4.8%; 12%) at 12-mo. All 30 patients were available for the BL examination as well as for the 6-mo and 12-mo follow-up examinations. Thus, the recall rate was 100% at all time points. Figure 1 shows the flow of participants through this study up to 12-mo in accordance with the CONSORT 2010 statement [32].

### Restoration characteristics

Restoration characteristics are summarized in Table 2. The main reason for restoration placement was secondary caries, followed by restoration replacement for other reasons (e.g., fracture), and primary caries. All teeth were sensitive to cold prior to restoration. Restorations were evenly distributed with respect to location. In the FOBF group, restoration size was limited to two- or three-surface-restorations. In the SABF group, restoration size included mainly two-surface-restorations, but also three- and four-surface-restorations. The latter were mesial-occlusal-distal restorations with buccal or oral extensions, but without any cusp replacements. Indirect pulp capping was performed in 11 cases in the FOBF group and in 12 cases in the SABF group. Restoration margins in the proximal box were located within enamel in 22 restorations in each group and were extended to the dentin in 8 restorations in each group. In the FOBF group, 7 restorations had one proximal dentin margin, and one restoration had two proximal dentin margins. In the SABF group, 8 restorations had one dentin margin.

**Table 2:** Restoration characteristics

		FOBF	SABF
Reason for restoration	Primary caries	3	6
	Secondary caries	17	16
	Restoration replacement for other reasons	10	8
Location	Upper jaw premolars	9	6
	Upper jaw molars	9	11
	Lower jaw premolars	6	6
	Lower jaw molars	6	7
Surfaces	2	17	23
	3	13	5
	≥ 4	0	2
Indirect pulp capping	Yes	11	12
	No	19	18
Dentin margins	No	21	22
	1	7	8
	2	1	0
Shade	A2	8	7
	A3	19	18
	B1	2	3
	B2	-	1

Restoration characteristics of FOBF and SABF restorations

### Clinical performance according to selected FDI criteria

#### Dissents among examiners

Among all evaluated FDI criteria in all 30 patients with two restorations each, there were 4.4% dissents between both examiners at baseline, 4.5% at 6-mo and 4.6% at 12-mo, which were immediately resolved by discussion between both examiners, while the patient still was present and could be jointly re-evaluated by both examiners.

#### Esthetic properties

Table 3 shows the clinical data of all pairs of restorations at all examination time points (BL, 6-mo, 12-mo) for selected criteria from the esthetic properties panel. There were only clinically acceptable scores (scores 1–3) for both materials at all examination time points.

**Table 3:**
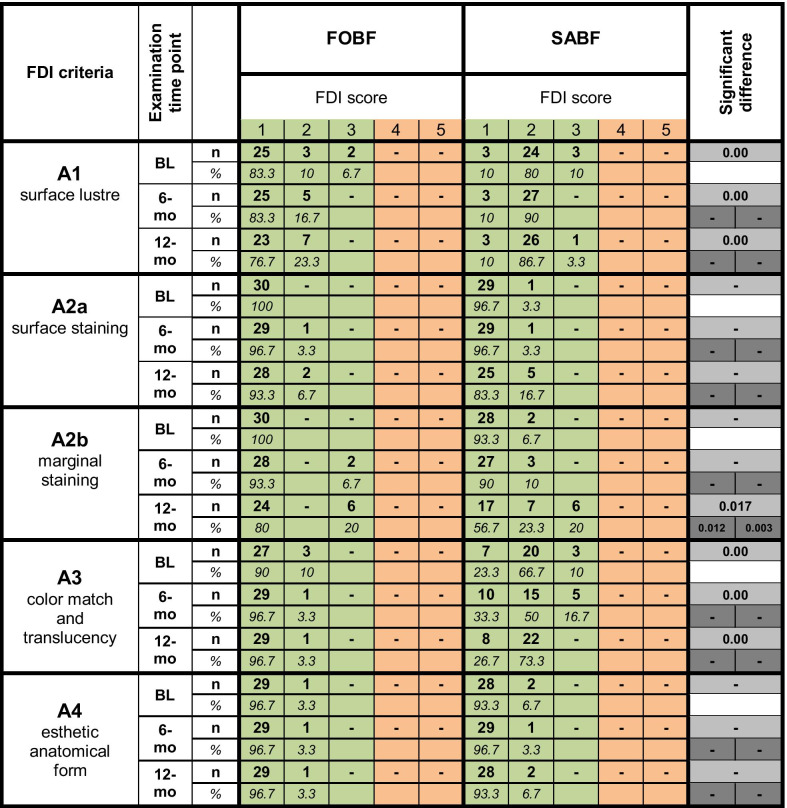
Clinical data for esthetic properties according to FDI criteria. Frequencies of FDI scores 1–5 (number of restorations (*n*) and percentages (%)) are depicted for FOBF and SABF. Clinically acceptable scores (1–3) are highlighted in green, non-acceptable scores are highlighted in orange. *p*-values show significant differences between materials at a respective examination time point in light grey, and significant differences within a material over time (BL vs. 6 month, BL vs. 12 month) in dark grey (FOBF left, SABF right)

**Fig. 2:**
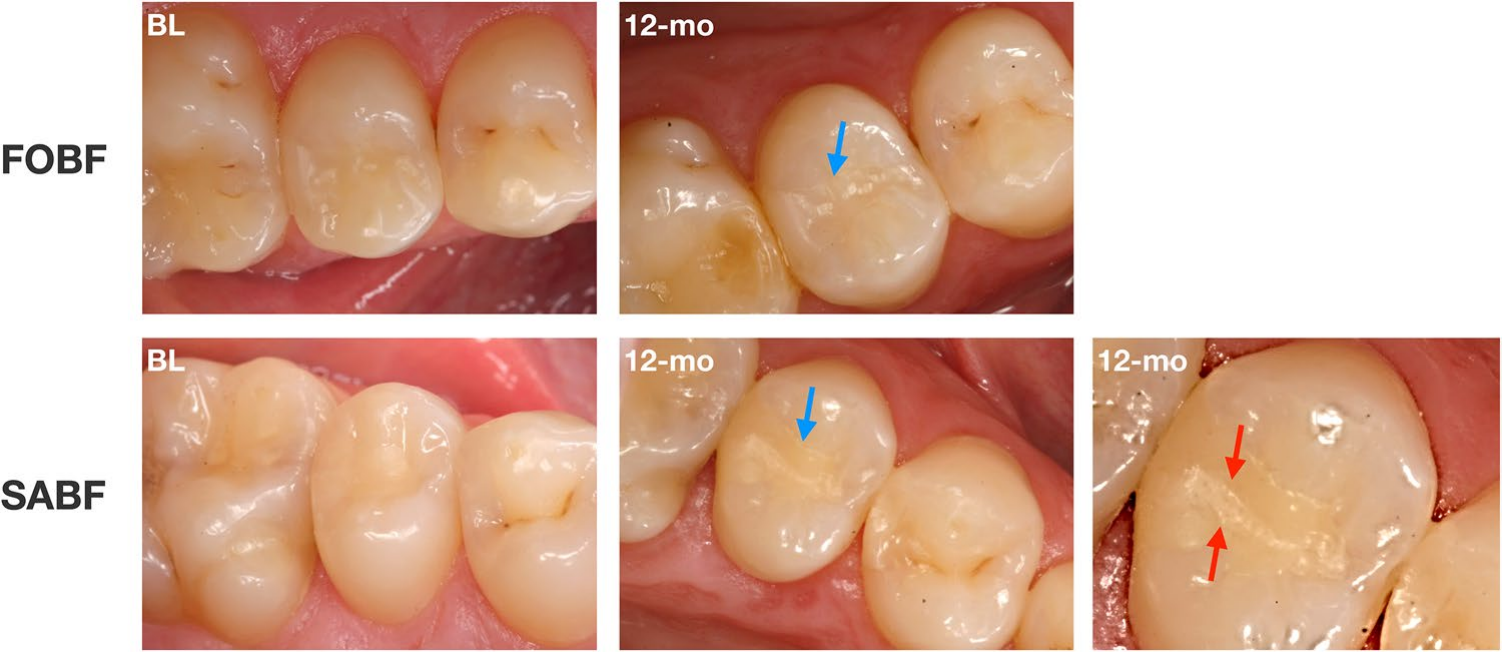
Exemplary depiction of differences in *surface luster* between both materials over time. Top row: occlusal-distal FOBF restoration on tooth 25 at BL and 12-mo. Bottom row: Occlusal-distal SABF restoration on tooth 15 at BL and 12-mo. Note the differences in surface lustre (indicated by blue arrows) and the isolated pores in SABF (indicated by red arrows)

For *surface lustre* (A1), materials were found to be significantly different (*p* = 0.00) at all examination time points, with FOBF mainly showing clinically excellent luster similar to enamel (score 1; BL: 83.3%; 6-mo: 83.3%; 12-mo: 76.7%), whereas SABF predominantly exhibited slightly dull surfaces with isolated pores (score 2; BL: 80%, 6-mo: 90%, 12-mo: 86.7%) or dull but acceptable surfaces (score 3; BL: 10%; 12-mo: 3.3%). Figure 2 shows clinical examples for both materials.

Regarding *surface staining* (A2a), no significant differences were recorded between both materials. At 12-mo, SABF revealed the highest overall level of surface staining, considered to be easily removable by polishing (score 2) in 16.7% of the restorations.

**Fig. 3:**
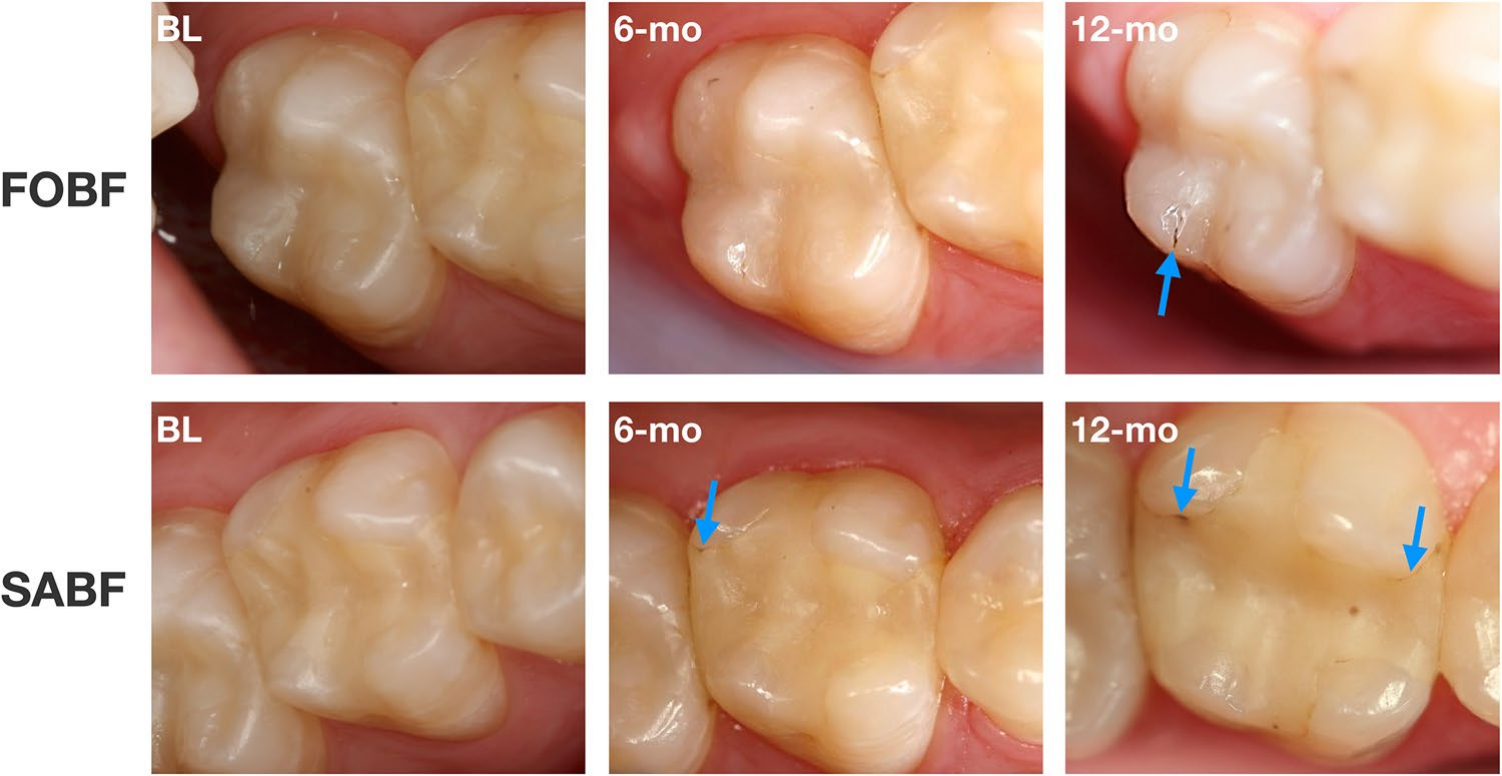
Exemplary depiction of differences in *marginal staining* between both materials over time. Top row: Mesial-occlusal FOBF restoration on tooth 27 at BL, 6-mo and 12-mo. Bottom row: mesial-occlusal-distal SABF restoration on tooth 26 at BL, 6-mo and 12-mo. Note the increasing marginal staining in FOBF at 12-mo and in SABF at 6-mo and 12-mo (indicated by blue arrows)

With respect to *marginal staining* (A2b), there was a significant difference between both materials at 12-mo with SABF showing significantly more (*p* = 0.017) marginal staining as compared to FOBF. Furthermore, both materials revealed a significant increase in marginal staining over time (SABF: *p* = 0.003; FOBF: *p* = 0.012). Figure 3 shows clinical examples for both materials.

**Fig. 4:**
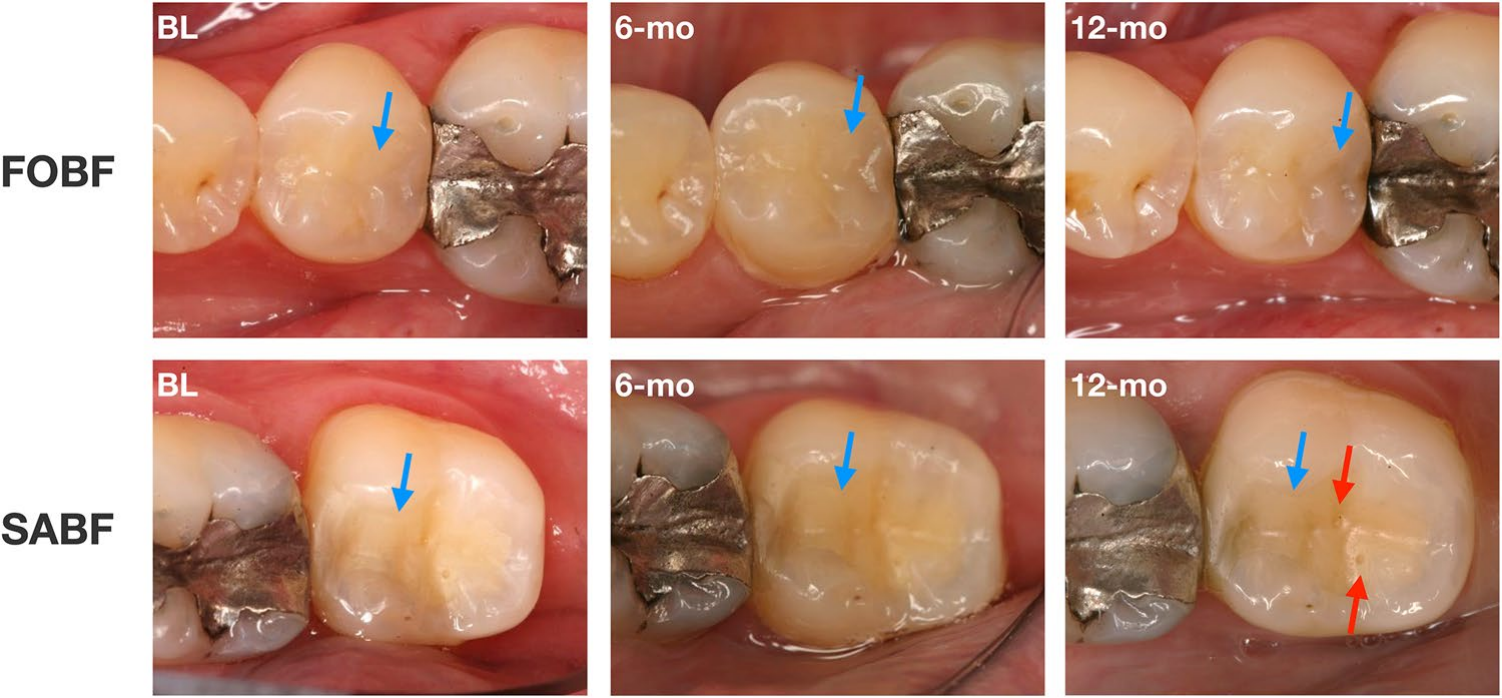
Exemplary depiction of differences in *color match and translucency* between both materials over time. Top row: occlusal-distal FOBF restoration on tooth 35 at BL, 6-mo and 12-mo. Bottom row: Mesial-occlusal SABF restoration on tooth 37 at BL, 6-mo and 12-mo. Note the differences in color match and translucency between FOBF and SABF (indicated by blue arrows; SABF more yellowish and opaque) as well as the isolated pores in SABF (indicated by red arrows)

FOBF yielded significantly better *color match and translucency* (A3) than SABF at all examination time points (*p* = 0.00). While FOBF restorations showed good color match without difference in shade or translucency (score 1; BL: 90%; 6-mo: 96.7%; 12-mo: 96.7%) at all examination time points, SABF revealed predominantly minor deviations in color match (score 2; BL: 66.7%; 6-mo: 50%; 12-mo: 73.3%) or distinct but acceptable deviations (score 3; BL: 10%; 6-mo: 16.7%). Figure 4 shows clinical examples for both materials.

Regarding *esthetic anatomical form* (A4), there were no significant differences between both materials or over time. Both SABF and FOBF yielded an ideal form (score 1) in more than 93% of the restorations irrespective of the examination time point.

#### Functional properties

Table 4 shows the clinical data of all pairs of restorations at the distinct examination time points (BL, 6-mo, 12-mo) for selected criteria from the FDI functional properties panel. There were only clinically acceptable scores (scores 1–3) for both materials at all examination time points.

**Table 4:**
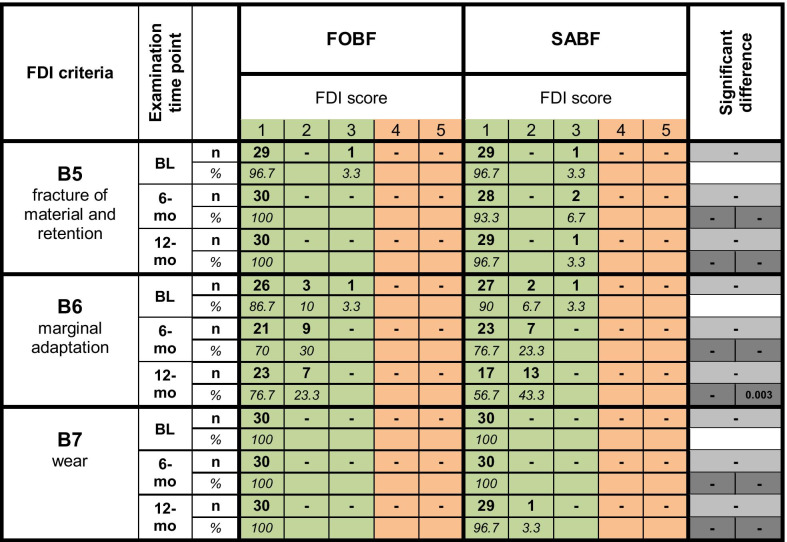
Clinical data for selected functional properties according to FDI criteria. Frequencies of FDI scores 1–5 (number of restorations (*n*) and percentages (%)) are depicted for FOBF and SABF. Clinically acceptable scores (1–3) are highlighted in green, non-acceptable scores are highlighted in orange. *p*-values show significant differences between materials at a respective examination time point in light grey, and significant differences within a material over time (BL vs. 6 month, BL vs. 12 month) in dark grey (FOBF left, SABF right)

With respect to *fracture of material and retention* (B5), no significant differences were recorded. At all examination time points, FOBF and SABF showed no fractures or cracks of material (score 1) in more than 93% of the restorations.

Regarding *marginal adaptation* (B6), there were no statistically significant differences between both materials. However, SABF revealed a significant increase (*p* = 0.003) in minor marginal irregularities, which was represented by an increase in slight ditching or slight steps or flashes (score 2) from 6.7% at BL to 43.3% at 12-mo.

Both materials exhibited *occlusal contour and wear* (B7) equivalent to enamel without any significant differences between materials or significant changes over time.

#### Biological properties

Table 5 shows the clinical data of all pairs of restorations at the distinct examination time points (BL, 6-mo, 12-mo) for selected criteria from the FDI biological properties panel. There were only clinically acceptable scores (scores 1–3) for both materials at all examination time points.

**Table 5:**
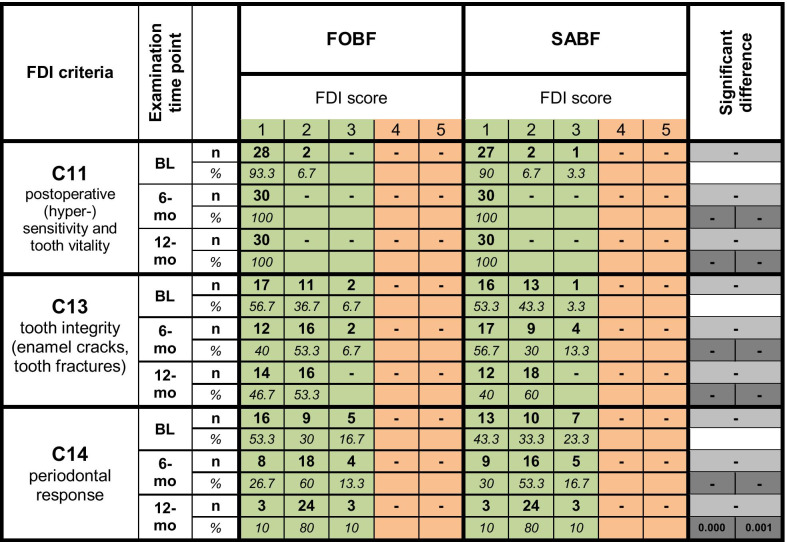
Clinical data for selected biological properties according to FDI criteria. Frequencies of FDI scores 1–5 (number of restorations (*n*) and percentages (%)) are depicted for FOBF and SABF. Clinically acceptable scores (1–3) are highlighted in green, non-acceptable scores are highlighted in orange. *p*-values show significant differences between materials at a respective examination time point in light grey, and significant differences within a material over time (BL vs. 6 month, BL vs. 12 month) in dark grey (FOBF left, SABF right)

For *postoperative (hyper-)sensitivity and tooth vitality* (C11), there were no statistically significant differences. At the 12-mo examination time point, all FOBF and SABF restorations revealed regular tooth vitality and no hypersensitivity.

With regard to *tooth integrity (enamel cracks, tooth fractures)* (C13), no significant differences were recorded between materials or over time. Hairline cracks in enamel (score 2) were detected at similar frequencies for FOBF (BL: 36.7%, 6-mo: 53.3%, 12-mo: 53.3%) and SABF (BL: 43.3%, 6-mo: 30%, 12-mo: 60%).

There were no significant differences in *periodontal response* (C14) between both materials. However, both materials exhibited a decrease in score 1 ratings (little plaque, no inflammation) and an increase in score 2 ratings (gingivitis, no pocket development) between BL and 12-mo over time, which was found statistically significant (FOBF: *p* = 0.000; SABF: *p* = 0.001).

## Discussion

### Study design

The present study investigated the clinical success and performance of a novel self-adhesive bulk-fill restorative material (SABF) and a conventional bulk-fill RBC (FOBF) applied in combination with a universal adhesive (SBU) in self-etch mode for restoration of class II cavities in premolars and molars. It was performed as a prospective, controlled, randomized clinical trial in 30 patients in a split-mouth design with two restorations in each patient, following the requirements outlined in the CONSORT 2010 statement [32]. The split-mouth design is regarded more favorable than a parallel design for clinical evaluation of restorative materials because patient-related aspects such as individual level of oral hygiene, diet, and habits like smoking or teeth grinding, which generally influence longevity and clinical performance of dental restorations, affect both groups equally [34]. The sample size of this study was calculated to be 26 patients, and 30 patients were recruited to compensate for potential dropouts in future follow-ups of this clinical trial. This sample size also clearly outnumbers the requirements defined in the former ADA acceptance guidelines for posterior restorations (i.e., split-mouth design with at least 20 patients with two restorations each) [35].

As outlined above, the scope of this study was to evaluate the novel restorative material SABF which may serve as an alternative to amalgam in terms of cost-effectiveness and ease of handling. SABF was applied without any pretreatment of dentin or enamel, while FOBF was used in combination with SBU in self-etch mode. Selective enamel etching was not performed in order to reduce the working steps.

The clinical examination of the restorations was based on the FDI clinical criteria and scoring system [29, 30], which is known to be more sensitive and discriminative than USPHS criteria for detecting early deterioration and sign of failures, especially regarding the criteria *marginal staining* and *marginal adaptation* [36–39]. All FDI criteria were examined for each restoration at each time point but only those criteria are included in the present study, which were considered to be relevant for assessing clinical performance of class II restorations after 12-mo of clinical service. Due to the complexity of the FDI clinical criteria and scoring system, most studies only report selected criteria according to the type and the aims of the respective study [31, 37, 38, 40, 41], which is in line with the recommendations by Hickel et al. [29], and was also summarized in a recent systematic review on the use of FDI criteria in clinical trials on direct dental restorations [36].

### Clinical performance according to selected FDI criteria

For the present study only those FDI criteria are reported which were considered to have meaningful relevance for the evaluation of restorations in class II cavities after 12-mo of clinical service (criteria A1, A2a, A2b, A3, A4, B5, B6, B7, C11, C13, C14). All other criteria not specifically mentioned here did not yield any conspicuities or significant differences between both restorative materials or over time. Both restorative materials exhibited clinically acceptable scores (i.e., scores 1–3) in all examined FDI criteria, accounting for a clinical success rate of 100% after up to 12-mo of clinical service.

#### Esthetic properties

*Surface lustre* was found to be significantly inferior for SABF as compared to FOBF at all examination time points. Surface polishing was compromised with SABF, which may be to some extent attributed to the composition of the material itself, and to the presence of small porosities and voids due to the mixing procedure of this two-component material. This may account for the predominantly dull *surface lustre* associated with the presence of isolated or multiple pores found in the SABF restorations. Despite the appearance of pores in SABF, *surface staining* was not an issue with either material after up to 12-mo with no significant differences between both materials. Both materials exhibited significantly increasing *marginal staining* over time (BL to 12-mo), which was more pronounced in the SABF restorations yielding a significant difference between SABF and FOBF at 12-mo. The occurrence of such slight marginal discolorations may be attributed to the lack of enamel etching. The inferior etching patterns of a mild universal adhesive like SBU (pH 2.7 [42]) or a self-adhesive RBC like SABF as compared to phosphoric acid may yield a less intimate bond and favor small imperfections at the enamel margins causing marginal discolorations during clinical service [43]. However, minor staining at either surfaces or margins can usually be easily removed by re-polishing, as it has been recommended to be performed at each recall appointment for ensuring longevity of dental restorations [31, 44]. Due to the lack of enamel etching and in view of using a novel self-adhesive restorative material, separate evaluation of enamel and dentin margins in terms of *marginal staining* and *marginal adaptation* may be of interest, as it has been discussed in a previous study on the clinical performance of flowable RBCs for restoration of NCCLs as a potential refinement of the original FDI criteria [31]. However, in class II cavities dentin margins, if present, are usually located in the depth of the proximal box and thus difficult to access for such a distinctive evaluation.

*Color match and translucency* were found significantly less suitable in SABF than in FOBF restorations at each examination time point, but within the clinically acceptable range (scores 1 to 3). FOBF restorations mainly showed perfect *color match and translucency*, whereas SABF restorations revealed minor deviations, mostly with regard to lower translucency. These differences may possibly be attributed to the general composition (e.g., with regard to filler particles) of both materials, or to intrinsic pores in the SABF restorative material, which may change light transmission and result in a slightly more opaque or darker appearance.

*Esthetic anatomical forms* could be achieved with both restorative materials, but with different approaches: FOBF could be sculpted and shaped prior to polymerization in order to obtain the desired tooth morphology. On the contrary, SABF needed be overfilled to a certain extent and the final restoration morphology was mainly achieved after polymerization by subtractive finishing and polishing procedures, whereas actual “sculpting” of the material in the unpolymerized condition was not possible.

#### Functional properties

With respect to *fracture of material and retention*, both materials performed similar up to 12-mo without any occurrence of bulk fractures or “catastrophic” failures in either material, accounting for sufficient mechanical properties of both materials. These features are also reflected in the data recorded for *occlusal contour and wear*, which indicates wear resistance, especially in more extended cavities.

There was a significant decrease in *marginal adaptation* in SABF restorations over time (BL to 12-mo), as represented by slight ditching, steps and flashes or minor irregularities, which was not recorded for FOBF. This corresponds to the significantly higher marginal discoloration found for SABF at 12-mo discussed above. Increasing marginal deterioration is usually accompanied by occurrence of marginal discolorations which may indicate degradation of the adhesive interface associated with wear at this interface and formation of small marginal gaps [45, 46]. Despite this significant difference in marginal adaptation over time recorded for SABF, all restorations of both materials were scored within FDI scores 1 and 2 only at 12-mo, representing clinically very good to good ratings and thus fully acceptable restorations. Therefore, from an overall point of view, the recorded change in marginal integrity from clinically very good to clinically good may be significant but does not seem to be critical in terms of clinical material performance.

#### Biological properties

*Postoperative (hyper-)sensitivity* was detected in two FOBF and three SABF restorations at BL, but within a clinically acceptable range (scores 2 and 3). These postoperative hypersensitivities had ceased prior to the 6-mo evaluation. Previous clinical studies have also reported occurrence of hypersensitivities during the first days or weeks after placement of the restoration, which usually settled within a short time [47, 48]. Therefore, the rather mild and transient cases of hypersensitivity observed here are rather attributed to the restorative procedures (e.g., caries excavation, placement of rubber dam, drying) than to the respective restorative materials [47]. This wide-scale absence of postoperative hypersensitivities may also hint to sufficient self-adhesive properties of SABF and to sufficient curing depth of both restorative materials even in the deeper layers of the restorations.

Assessing *tooth integrity (enamel cracks, tooth fractures)* over time is important for clinical evaluation of novel dental restorative materials. Particularly, in case of SABF which contains acid-reactive glass fillers in its powder part, it is crucial to exclude occurrence of enamel fractures due to water uptake and spatial expansion, as it had been observed for restorative materials previously [49–52]. About half of the teeth restored in this study revealed hairline cracks, irrespective of the material and the examination time point but significant deterioration in tooth integrity was not an issue with either material.

With regard to *periodontal response*, there was a significant decline over time for both materials between BL and 12-mo, but within a clinically fully acceptable range. In this context, it must be considered that this criterion just records a brief and local PBI “snapshot” as compared to the BL situation and to a control tooth. On the contrary, the median full-mouth PBI values even slightly decreased from 8.5% at BL to 7.5% at 12-mo, representing an overall very good oral hygiene level of the patient cohort. In the course of this study, it became obvious that the original instructions for using the criterion *periodontal response* (C14) can be misleading. According to these instructions, an increase up to one grade in severity of PBI compared to BL or to a control tooth should be rated as score 3 (clinically acceptable), while an increase of more than one PBI-grade with the need of intervention should be rated as score 4 (not clinically acceptable). Accordingly, there is no instruction on how to proceed with clinically not relevant local PBI fluctuations without any need of intervention, e.g., a temporary increase of two PBI grades from grade 0 to grade 2. Therefore, interpretation of this criterion was amended for this study by discerning scores 3 and 4 just with respect whether there was a need of intervention, e.g., in terms of recontouring the cervical aspect of a given restoration due to overhangs.

In summary, the null-hypothesis of this study could not be rejected: both restorative materials exhibited only clinically acceptable scores in all examined FDI criteria. FOBF and SABF exhibited similar clinical performance in functional and biological properties, but FOBF showed significantly better performance with regard to esthetic properties *surface lustre* and *color match and translucency* at all examination time points and *marginal staining* at 12-mo than SABF. These differences in esthetic properties were already observed at BL and did not intensify over time up to 12-mo of clinical observation. Therefore, SABF seems to be a slightly less esthetic restorative material as compared to FOBF. Within the limitations of this study, the novel self-adhesive bulk-fill restorative SABF (as well as the bulk-fill RBC FOBF) showed promising results and thus may be recommended for clinical use. However, it is of vital importance to perform further follow-ups of this clinical trial in order to evaluate the clinical performance of SABF in the long term.
